# Needleless Melt-Electrospinning of Biodegradable Poly(Lactic Acid) Ultrafine Fibers for the Removal of Oil from Water

**DOI:** 10.3390/polym9020003

**Published:** 2017-01-25

**Authors:** Haoyi Li, Yi Li, Weimin Yang, Lisheng Cheng, Jing Tan

**Affiliations:** 1 College of Mechanical and Electrical Engineering, Beijing University of Chemical Technology, Beijing 100029, China; lihaoyi-hoy@163.com (H.L.); 18101287383@163.com (Y.L.); angwm@mail.buct.edu.cn (W.Y.); chengls@mail.buct.edu.cn (L.C.); 2 State Key Laboratory of Organic-Inorganic Composites, Beijing University of Chemical Technology, Beijing 100029, China; 3 Beijing Advanced Innovation Center for Soft Matter Science and Technology, Beijing University of Chemical Technology, Beijing 100029, China

**Keywords:** poly(lactic acid), needleless melt-electrospinning, oil sorption

## Abstract

As environmentally friendly and degradable material, Poly(lactic acid) (PLA) ultrafine fibers are promising candidates for the removal of oil from water. In this work, a self-established needleless melt-electrospinning process was used to produce PLA ultrafine fibers with diameters in the range of 800 nm–9 µm. In order to obtain ultrafine fibers, three types of hyperbranched polymers were respectively added into the melt for electrospinning. Effects of amount and molecular weight of the added hyperbranched polymers on average fiber diameter and its distribution, and contact angle were investigated. The prepared PLA ultrafine fibers exhibited superhydrophobicity with the contact angle as high as 156°, making it a potential candidate in marine oil spill recovery. The oil sorption capability of these fibers is as high as 159, 118, and 96 g/g for motor oil, crude oil, and diesel, respectively. Even after seven cycles of reuse, the fiber still maintained about 60% of its initial capacity of sorption. The kinetics of oil sorption in the film agrees very well with the pseudo-second-order kinetic model. This work is expected to promote the mass production and application of biodegradable PLA fibers in the treatment of marine oil spill pollution.

## 1. Introduction

In accidents or deliberate actions, heavy crude oil would be inevitably spilled into the environment—such as oceans, lakes, and rivers—which is not only a loss of oil but also a disastrous damage to the aquatic life and ecological system [1,2,3]. Thus, practically applicable and environmentally friendly methods or materials are in great need for the recovery of water polluted by spilled oil.

General approaches for the treatment of oil spills could be classified as physical, chemical, and biological methods among which physical sorption of spilled oil by adsorbent is the most economical and efficient one [4,5,6]. The ideal oil sorbents should be highly hydrophobic, which promises the capabilities of a large amount and high rate of oil sorption. For environmental protection purposes, the sorbents need to be reusable, collectable, and biodegradable [7,8]. In addition, they should also be buoyant and durable in aqueous media. As for materials for oil sorption, recent efforts are mainly focused on natural sorbents, high oil sorption resin, and polymer ultrafine fibers [4,9,10]. Natural sorbents—such as kapok, milkweed, and bagasse—have drawn worldwide research interests owing to their porous structure, wide availability, and biodegradability [2,11]. However, most of these sorbents are hydrophilic and of weak capacity of oil sorption, which to a large degree restrains their applications in oil spill recovery [12]. With the development of nanotechnology, the fabrication of ultrafine polymer fibers drives new applications in many areas such as air filtration, biomedicine, as well as water purification [13,14]. It is also possible to apply hydrophobic and oleophilic ultrafine polymer fibers in the removal of oil spills. Micro- or nano-scale polymer fibers are featured for their being highly porous, having a large surface-to-volume ratio, and superior mechanical performance, which gives them extremely high oil sorption capacity and outstanding reusability.

As a versatile technique for continuous preparation of ultrafine fibers, electrospinning has been extensively studied in academia and industry in recent years [15,16]. Zhu et al. [17] first applied solution electrospinning to the fabrication of oil sorbent for the separation of oil from seawater in 2011. It was found that PVC (Polyvinyl chloride)/PS (Polystyrene) fibers with a diameter of 1.5–3 μm and a porosity of 99.7% exhibit an oil sorption of 146 g/g for motor oil. Wu et al. [18] later indicated that porous PS fibers with a diameter of 0.540 μm and a total pore volume of 2.145 ± 0.032 mL/g exhibit an oil sorption of 131.63 g/g for motor oil. Lin et al. [19] systematically investigated the feasibility of the one-step solution electrospun nanoporous PS fibers as oil sorbents in terms of oil sorption. They also aimed to increase the reusability of electrospun fibers by various techniques, such as multi-nozzle electrospinning and co-axial electrospinning. They found that a comparable oil sorption capacity was able to be maintained even after the electrospun fibers of commercial PP (Polypropylene) were reused for several times [19,20,21]. However, all above-mentioned electrospun fibers are non-biodegradable, and most of the used sorbents would end up as landfills and incineration, which will cause secondary pollution to the environment. To the best of our knowledge, no work has been reported on the melt electrospinning of biodegradable ultrafine fibers for the removal of marine oil spills.

Compared with solution electrospinning, melt electrospinning has advantages, such as high throughput, smooth surface of resultant fiber, and absence of solvents [22]. It is especially suitable for the production of thermoplastic polymer fibers, such as PP and polylactic acid (PLA) [23]. PLA is termed an “environmentally-friendly material of the 21st century”, since the degradation products of PLA are mainly water and carbon dioxide, which are nontoxic to the environment [24].

In this work, PLA ultrafine fibrous mat was fabricated with self-established needleless melt electrospinning device, which is potentially able to achieve mass production of ultrafine fibers by a single nozzle. The behavior and mechanism of oil sorption of the prepared fibers were also discussed.

## 2. Experiments

### 2.1. Materials

PLA pellets (molecular weight is 70,000; glass transition temperature is 56 °C; melting point is 150 °C; thermal decomposition temperature is 250–300 °C) were procured from Shenzhen Guanghua Weiye Industrial Co., Ltd. (Shenzhen, China). Before melt electrospinning, the PLA pellets were dried for 10 h at 60 °C.

In order to obtain ultrafine PLA fibers, three types of hyperbranched polymers (HBPs) —namely H201, H202, and H203—were introduced to modify the PLA melts. These HBPs are hydroxy terminated, and procured from Suzhou HyPerT Resin Science & Technology Co., Ltd. (Suzhou, China). Their properties are listed in Table 1.

Three types of oil namely motor oil, crude oil, and diesel were selected to investigate the sorption characteristics of the fibrous sorbents. The properties of the tested oils are listed in Table 2. The viscosity of the tested oil was measured by a NDJ digital viscometer (Shanghai Jingtian Electronic Instrument Co., Ltd., Shanghai, China) at room temperature and shear rate of 200 s^−1^.

### 2.2. Melt Differential Electrospinning Eechnique

**Figure 1:**
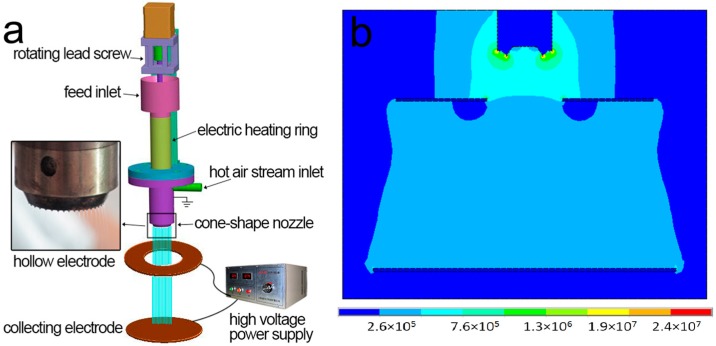
Schematic diagram (**a**) and electric simulation (**b**) of melt differential electrospinning device.

Figure 1a shows the schematic illustration of the self-designed melt differential electrospinning device (Beijing Weihuasheng Co., Beijing, China), which is composed of seven major components: feed inlet, high voltage power supply, hollow electrode, collecting electrode, heating rings, and a cone-shape nozzle with a hot air stream inlet. Compared with conventional melt electrospinning devices, this device has three major innovative points. First, a round distribution of Taylor cones can be formed along the bottom edge of the nozzle in electrostatic fields, which tremendously improves electrospinning productivity. Second, a lower voltage is applied to the hollow electrode and a higher one is applied to the collecting electrode, which generates a coupling electric field as simulated by the ANSYS software in Figure 1b. Third, a hot air stream is ejected through the middle of the nozzle, which can not only enhance the stretching of the jet, but also maintain its temperature and delay its solidification. As a result, ultrafine fibers could be prepared. During electrospinning, the dried PLA pellets were mixed with HBPs in ratios of 0%, 4%, 8%, and 12% by weight. Afterwards, these 10 mixtures were electrospun under the same processing conditions and 10 fiber samples would be obtained, namely, PLA, (4% H201)/PLA, (8% H201)/PLA, (12% H201)/PLA, (4% H202)/PLA, (8% H202)/PLA, (12% H202)/PLA, (4% H203)/PLA, (8% H203)/PLA, and (12% H203)/PLA. The temperature of polymer melt was set as 230 °C. The distances between the cone-shape nozzle and the hollow electrode—and between the hollow electrode and the collecting electrode—were set as 30 and 110 mm, respectively. High voltages of 30 and 65 kV were applied to the hollow electrode and the collecting electrode, respectively. The rotating speed of the leading screw was set as 20 r/min. All the experiments were conducted at room temperature of 25 ± 3 °C and humidity of 30%.

### 2.3. Characterization

The diameter of the electrospun fiber was imaged using a scanning electron microscopy (SEM, Hitachi Ltd., Tokyo, Japan) with an electron beam of 15 kV. Prior to their being observed, all fibers were coated with a 10 nm layer of platinum. The average fiber diameter and standard deviations from 50 fibers of each sample were determined with the open source image processing software, Image J (National Institutes of Health, Bethesda, MD, USA).

The wettability of electrospun fibers by water and motor oil was measured by a contact-angle apparatus (Dataphysics, Filderstadt, Germany). Before observation, the fluffy fibrous mat was flattened on a slide, and the average contact angle was measured and averaged over different positions of the same sample.

The thermal properties of the fabricated fibers were analyzed by a differential scanning calorimetry (DSC, Q2000, Ta Instruments, New Castle, USA) at a heating rate of 10 °C/min from room temperature to 200 °C in nitrogen atmosphere. The crystalline phase and properties of the prepared fibers were evaluated by an X-ray diffractometer (XRD, Rigaku, Tokoy, Japan) through CuKα radiation scanning (40 kV, 40 mA) over the 2θ range of 3°–90° with a step of 0.02°.

### 2.4. Oil Sorption Test

To estimate the maximum oil sorption capacity of the electrospun fibers, sorption test was first conducted in a pure oil system by placing 1.00 g sorbent into 300 mL oil in a 500 mL beaker at 30 ± 2 °C. During the sorption process, the amount of adsorbed oil at different times was recorded to plot the curve of sorption kinetics. After sorption for 60 min, the wet sorbent was taken out and placed on a metal mesh for 2 min to let the absorbed oil drain from the fibers, and then was weighed. The oil sorption capacity was obtained according to the following expression:

(1) $$Q=\frac{m_{t}-\left(m_{i}+m_{w}\right)}{m_{i}}\times 100\%$$

where *Q* is the oil sorption capacity (g/g), *m*_i_ is the initial weight of the dry sample, *m*_t_ is the weight of wet sample (g), *m*_w_ is the weight of the adsorbed water (g). All sorption tests were independently repeated three times to calculate the average and standard deviation.

To determine the oil/water selectivity of sorption, tests were also carried out in an oil/water bath by placing 1.00 g sorbent into the mixture of 500 mL water and 300 mL oil in a 1 L beaker at room temperature. The adsorbed water was measured according to the distillation technique as described in ASTM D95-05 [25].

### 2.5. Test of Reusability and Recoverability

The oil-loaded fiber samples were moved out of the tested oil with a nipper and then the adsorbed oil would be extracted out by a sand core funnel for about 10 min. Oil can be recovered without severe disruption of the morphology and mechanical strength of the fibrous sorbent. Such a sorption/desorption process was repeated for six cycles to determine the reusability and recoverability of fibrous material.

## 3. Results and Discussion

### 3.1. Morphology Analysis

**Figure 2:**
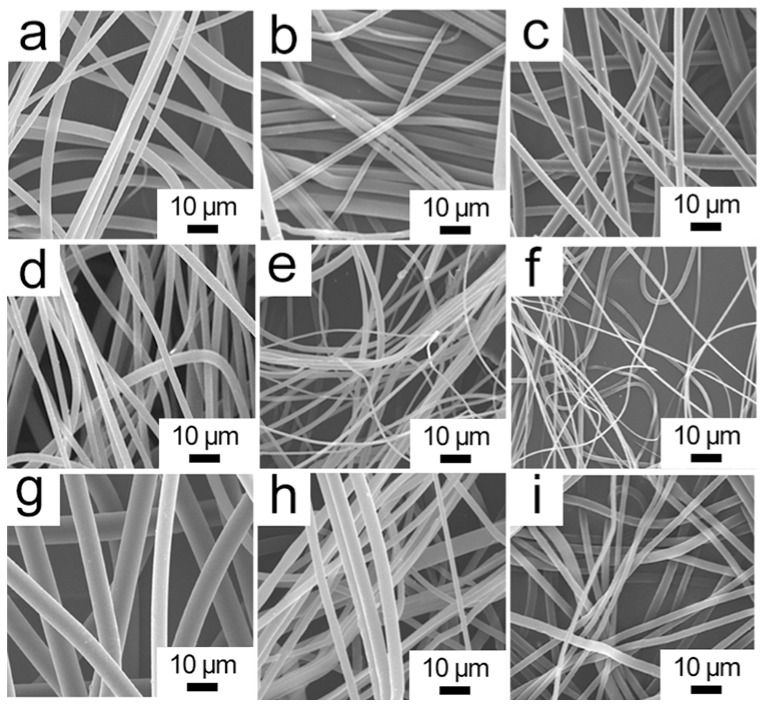
SEM images of melt electrospun fibers ((**a**–**i**) (4% H201)/PLA, (4% H202)/PLA, (4% H203)/PLA, (8% H201)/PLA, (8% H202)/PLA, (8% H203)/PLA, (12% H201)/PLA, (12% H202)/PLA, and (12% H203)/PLA.

**Figure 3:**
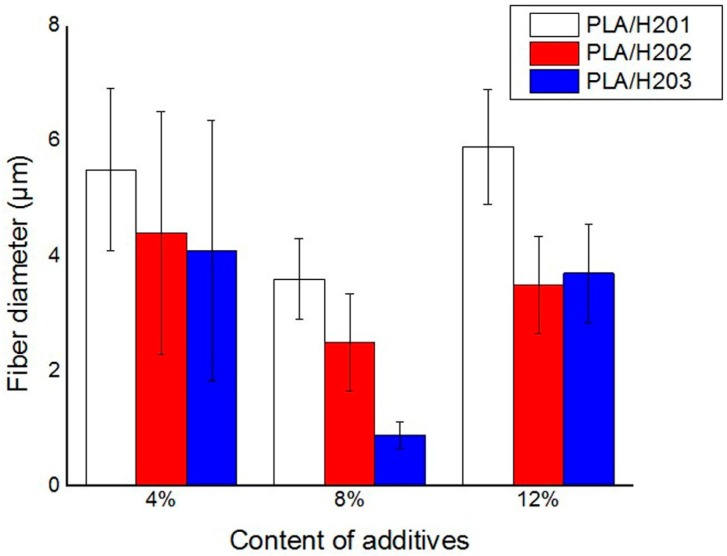
The relation between fiber diameter and the type and content of HBPs.

Figure 2 shows the morphology of the prepared fibers. The figure reveals that all melt electrospun fibers with different amounts of additives exhibit thread-like shape with smooth surface, and the porous and interconnected configuration could serve as oil storage vessel with huge volume. As shown in Figure 3, the presence of hyperbranched polymers has a significant effect on reducing the diameter of the electrospun fiber. All those PLA/HBP composite fibers are smaller than 6 µm. HBPs are highly branched with a large number of hydroxyl end groups, and generally appear as a compact and quasi-spherical shape. In melt electrospinning, external electrostatic forces are needed to disentangle the polymer chains from the stretched and entangled state. On the one hand, HBPs can function as lubricants and compatibilizing agents in the blend so that they can penetrate into entangled PLA molecules and thus reduce the entanglements of molecular chains. On the other hand, owing to the low viscosity of HBPs, they can serve as sliding balls and improve the slippage of the intertwined molecular chains, which could dramatically reduce the viscosity of the PLA melt and increase its fluidity. Under high voltage, a PLA melt with low viscosity can overcome surface tension and viscous resistance more easily. As a result, the polymer melt can be stretched into thinner fibers.

The component of HBPs plays a significant role in controlling the diameter of the final fiber. The addition of 8% H203, the prepared fibers have an optimized diameter of 896 nm, which is much smaller than that of the neat PLA fibers. This result may be ascribed to the fact that only part of PLA molecular chains are untangled when the content of HBPs is relatively low (4%), while the disentanglements of PLA polymer chains increased with HBPs concentration and nearly all chains were untangled when the HBP content reached 8%. However, the average fiber diameter increased when continuously increasing the HBPs to 12%. This may be due to the fact that a majority of chain entanglements have already been removed with the presence of 8% HBPs, and further increasing the additive content would not help in reducing fiber diameter. In addition, HBPs with a similar structure but different molecular weight and hydroxyl group number also have an evident impact on the diameter and distribution of PLA fiber. The averaged fiber diameter decreases when increasing the molecular weight of the added HBPs. This result is understandable, because HBPs with larger molecular weights can separate the PLA chains from each other with a larger distance, which results in a lower viscosity.

### 3.2. Contact Angle Analysis

**Figure 4:**
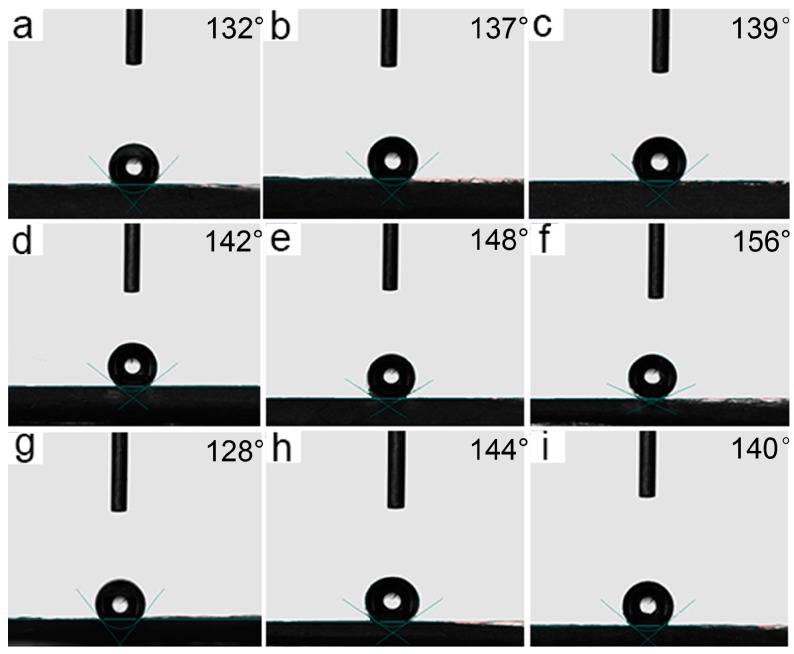
Contact angle images of melt electrospun fibers ((**a**–**i**) (4% H201)/PLA, (4% H202)/PLA, (4% H203)/PLA, (8% H201)/PLA, (8% H202)/PLA, (8% H203)/PLA, (12% H201)/PLA, (12% H202)/PLA, and (12% H203)/PLA.

Contact angle images of melt electrospun fibers are shown in Figure 4. According to SEM images, the decreasing of fiber diameter results in an increasing of water contact angle. Thinnest 8% H203/PLA fiber shows superhydrophobicity with a water contact angle of 156°. According to the Wenzel and Cassie-Baxter model, superhydrophobic surface can be prepared by either generating hierarchical micro- and nano-structures onto material surface to increase surface roughness or conducting hydrophobic modification to decrease surface energy [26]. Melt differential electrospinning has been proven to be a simple and cost-effective technique for the production of ultrathin fiber assembly [27]. Hydrophobic and oleophilic fibrous sorbent can be modeled as a capillary matrix of porous material.

The Young-Laplace equation proposed to describe liquid sorption into a single capillary tube in previous work can be used to estimate the capillary pressure that imbibes liquid into the inter-fiber pores [28].

(2) $$\Delta P=\frac{2\gamma \cos\theta }{r}$$

where Δ*P* is the capillary pressure, *r* is the representative radius of the capillary, γ is the liquid surface tension, and θ is the contact angle at the fiber-liquid interface along with the capillary.

Based on this equation, a contact angle of 90° is the critical point in deciding whether oil/water liquid can be absorbed or rejected by the capillary matrix. If an oil droplet is in contact with the surface of fibrous sorbent, it is first adsorbed by the Van Der Waals attraction, and then it penetrates into the pores of the inter-connected cylinder-shaped fibers by capillary forces. The tested contact angle of all eletrospun fibers for motor oil is 0°, suggesting that the highest capillary force of fibrous sorbent acts on motor oil. When θ > 90°, Δ*P* becomes negative, indicating a repelling force of ultrafine fibers exerted on water. The superhydrophobicity and superoleophilicity properties of electrospun fibrous sorbent could enable the efficient separation of oil from polluted sea area.

### 3.3. DSC and XRD Investigations

**Figure 5:**
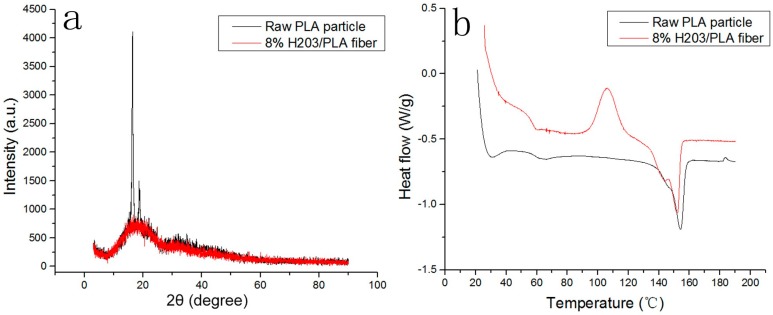
XRD scan (**a**) and DSC curve (**b**) for raw PLA particle and (8% H203)/PLA fiber.

As shown in Figure 5a, the raw PLA particles exhibit a clear and sharp crystalline peak at about 2θ = 17° due to a certain degree of isotactic formation of the molecular arrangement after the polymerization reaction of l-lactic acid. However, for the (8% H203)/PLA fiber, the crystalline peak disappears and becomes a relatively smooth and broad amorphous zone, which could be attributed to the mechanical and thermal degradation of the polymer chains caused by high processing temperature and strong screw scission.

The DSC thermograms of the tested materials are shown in Figure 5b. The presence of melting peaks at about 154.2 and 152.1 °C, respectively, for the raw PLA particle and the (8% H203)/PLA fiber indicates the existence of crystallinity in both materials. The decreasing melting peak of PLA after electrospinning may be attributed to the amorphous domains caused by thermal and mechanical decomposition impurities. The cold crystallization peak of the electrospun fiber at about 95 °C indicates that the crystallization of the PLA did not occur because of the quick cooling of electrospun fibers before being collected.

### 3.4. Oil Sorption Test in Pure Oil System

#### 3.4.1. Oil Sorption Experiments

**Figure 6:**
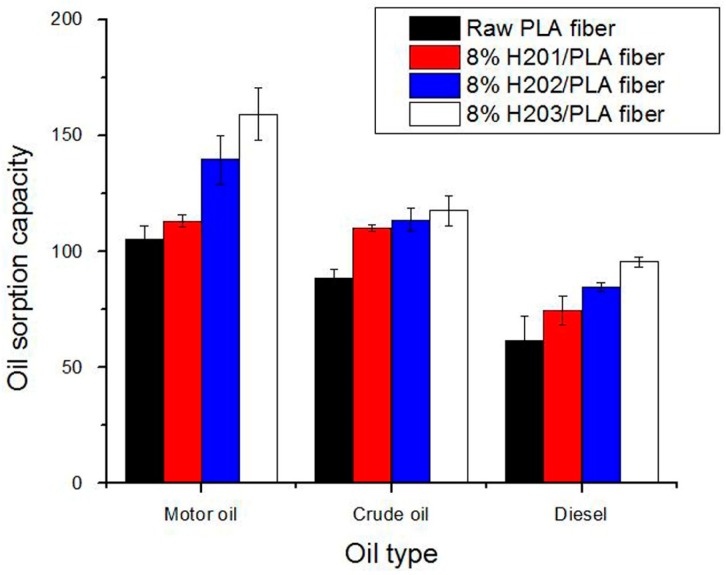
Oil sorption capacity of melt electrospun fibers for different types of oil.

The above-mentioned interaction between water/oil and electrospun fibrous membrane demonstrates that oil could be selectively absorbed by the electrospun fibers when the fibrous sorbent is in contact with oil-water mixtures. The oil sorption tests were first conducted in a pure oil system without any water to qualitatively evaluate the maximum oil sorption capacity for the raw and HBPs modified PLA fibers, respectively. As shown in Figure 6, the oil sorption capacity of the raw PLA fiber for the motor oil, crude oil and diesel is 105, 89, and 62 g/g, respectively, which is four to five times higher than that of the conventional PP sorbent. With the decreasing of fiber diameter, there is an apparent increase in oil sorption capacity. The (8% H203)/PLA fiber exhibits the highest oil sorption capacities of 159, 118, and 96 g/g for motor oil, crude oil and diesel, respectively. These obtained data suggests that fiber diameter plays an essential role in determining the oil sorption capacity of fibrous sorbent. Thinner fiber assembly has a larger specific surface area and higher porosity, so it provides larger area and space for absorbed oil to be wrapped into. It can also be observed that the oil sorption capacity of the fiber samples for the tested oils is in the order of motor oil > crude oil > diesel, which is related to the viscosity and density of the tested oils. The oil sorption capacity of the same fiber increases with the viscosity of the oil, because oil with a higher viscosity more easily adheres onto the fiber surface and retains in the inter-fiber voids. In the case of oil with low viscosity and low specific gravity, the oil flows into the capillary network of fibrous mats as well as onto the fiber surface at a certain speed, but it also desorbs easily at the same time. This finding is consistent with a report in previous literature [29].

#### 3.4.2. Reusability and Recoverability

**Figure 7:**
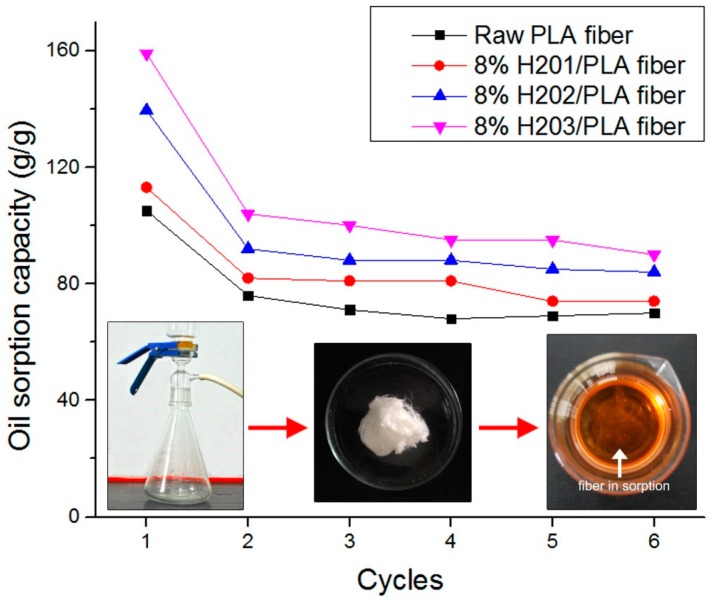
Oil sorption/desorption cycles of melt electrospun fibers.

After oil sorption, the oil molecules were trapped into the interconnected fiber assembly by the attracting Van Der Waals forces and the hydrophobic interaction between oil molecules and the hydrophobic groups on the fiber surface. Oil can be extracted from oil-swelled fiber by air pressure generated by a vacuum pump. The reusability of fibrous sorbent in term of oil sorption capacity for motor oil during six sorption/desorption cycles is shown in Figure 7. After the first sorption/desorption process, the fibrous sorbent cannot maintain its fluffy structure because of a lack of elasticity. Therefore, oil sorption capacity sharply decreases by about 27%–35% of its original at the second cycle, and 33%–43% approximately after six cycles. Nevertheless, its retained oil sorption capacity is still higher than that of most commercial PP oil sorbents. The sudden decrease of oil sorption capacity from the first to the second cycle is mainly attributed to the irreversible deformation or general deterioration of the fiber assembly due to strong mechanical pressure and residual oils trapped in the void of fibrous sorbent. On average, about 98% of the adsorbed oil could be recovered by the vacuum pump. The excellent reusability and recoverability of the electrospun fibers guarantees its practical applications in marine oil spill recovery.

### 3.5. Removal of Oil from Oil/Water Mixture System

**Figure 8:**
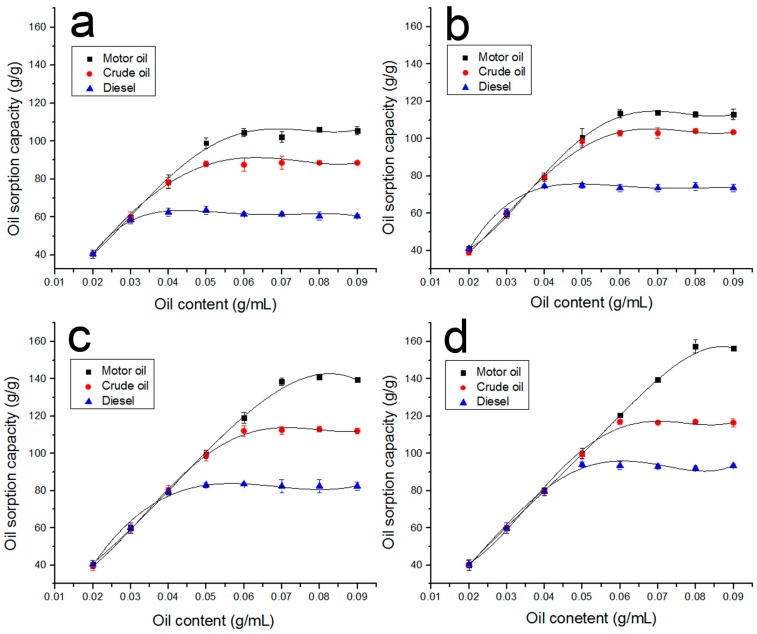
Effect of oil concentration on oil sorption capacity by raw PLA fiber (**a**); (8% H201)/PLA fiber (**b**); (8% H202)/PLA fiber (**c**); and (8% H203)/PLA fiber (**d**).

**Figure 9:**
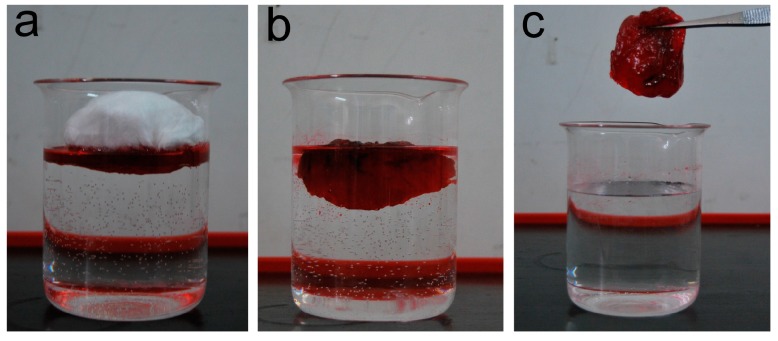
Optical images of recovery of motor oil (colored with oil red O) from water by raw PLA fiber. (**a**) Motor oil-water mixture; (**b**) oil being absorbed by fibrous sorbent; (**c**) complete separation of motor oil from water.

The oil sorption capacities of the raw and HBP-modified PLA fibers in oil/water mixture systems of various concentrations are shown in Figure 8. It is shown that the oil sorption capacities firstly increases with the amount of oil on the water’s surface and eventually reaches a sorption/desorption equilibrium. The maximum oil sorption capacity of raw PLA fiber for motor oil, crude oil, and diesel are approximately 103, 90, and 61 g/g, respectively. For the (8% H203)/PLA fiber, the oil sorption capacity is as high as 154, 115, and 98 g/g for above three oils, respectively. The little difference between these oil capacities in a pure oil system and an oil/water mixture system indicates that the outstanding oil/water selectivity of fibrous sorbent and the feasibility of the melt electrospun PLA fibers for the removal of marine oil spill. Figure 9 shows the optical images of motor oil recovery from water by the raw PLA fiber. At the initial stage of contact with oil, most of the floating oil was quickly sucked into the fiber assembly in a short time. Within less than 5 min, nearly all the oil was adsorbed. With excellent buoyance, the sorbent adsorbing oil still floated on the water’s surface during the whole process. After taking away the oil-loaded PLA fiber, the motor oil is removed completely from the water surface.

#### 3.5.1. Oil Sorption Kinetics

**Figure 10:**
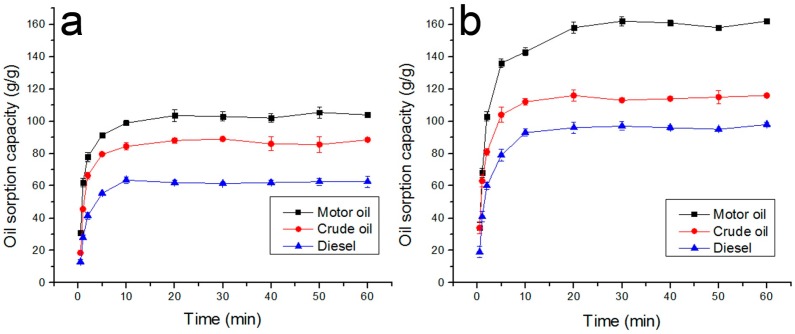
Adsorption kinetic curve ((**a**) raw PLA fiber; (**b**) (8% H203)/PLA fiber).

The oil sorption kinetics can describe the uptake rate and give the theoretical sorption capacity. To obtain the kinetic curve, the weight of the swollen sorbent was measured at different adsorption times (30 s, 1 min, 2 min, 5 min, 10 min, 20 min, 30 min, 40 min, 50 min, and 1 h) through the oil sorption process. As shown in Figure 10, the oil pickup rate for the tested oils is fastest and over 90% of the tested oils are absorbed in the first 5 min, and thereafter the pickup rate gradually slows down until the sorption-desorption process comes to an equilibrium state. The rapid sorption rate at the initial stage may be attributed to the huge area of available exterior surface and the strong affinity force between the fibrous sorbent and the oil molecules. Further on, the blockage of capillary tubes of fibrous sorbent gradually leads to the decrease of capillary numbers. Finally, a thermodynamic equilibrium is reached when the amount of oil desorbing form the fibrous sorbent is in dynamic equilibrium with the amount of oil being adsorbed onto the fibrous sorbent. The parameters of the oil sorption kinetics can be obtained by fitting the data of the oil sorption kinetic curve according to the Lagergren pseudo-first order and Lagergren pseudo-second order equations.

The pseudo-first order kinetics model given by Langergren and Svenska are expressed as

(3) $$\frac{dQ_{t}}{dt}=K_{1}(Q_{e}-Q_{t})$$

where *Q*_t_ (g/g) is the sorption capacity of swelling sorbent at time *t*, *Q*_e_ (g/g) is the oil sorption capacity at equilibrium, *K*_1_ (g/g·min^−1^) is the sorption rate constant. The integration of Equation (3) derives

(4) $$\ln(Q_{e}-Q_{t})=\ln Q_{e}-K_{1}t$$

**Figure 11:**
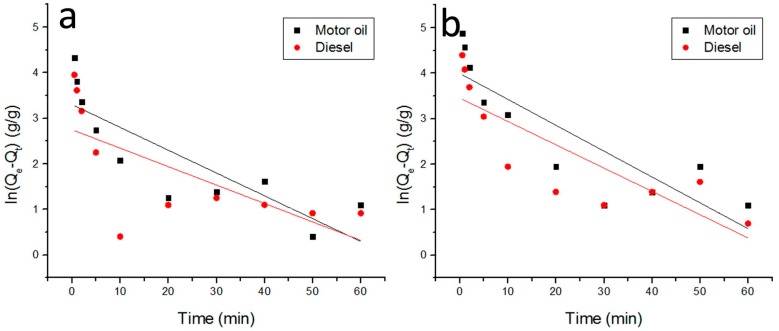
Linear fitting curve of first-order kinetic law of Lagergren model ((**a**) raw PLA fiber; (**b**) (8% H203)/PLA fiber).

The saturated sorption capacity *Q*_e_ and the sorption rate constant *K*_1_ can be calculated based on the slope of *K*_1_ and the intercept of ln*Q*_e_. The sorption time *T* for equilibrium can be obtained as the time for the sorption capacity to reach 95% of *Q*_e_. The linear fitting curves of the first-order kinetic law of the Lagergren model are shown in Figure 11. All the parameters of oil sorption kinetics are shown in Table 3. The determination coefficients *R*_1_^2^ range from 0.4204 to 0.7148, which are far less than 0.99, and there is an apparent inconsistency between the experimental sorption capacity and theoretical one. These results indicate that the sorption of oil onto the electrospun fibers does not follow the pseudo-first-order kinetic model.

The second order kinetics model is expressed as

(5) $$\frac{dQ_{t}}{dt}=K_{2}{\left(Q_{e}-Q_{t}\right)}^{2}$$

where *K*_2_ is the sorption rate constant (g/g·min^−1^). After the integration of Equation (5), we get:

(6) $$\frac{t}{Q_{t}}=\frac{1}{K_{2}{Q_{e}}^{2}}+\frac{t}{Q_{e}}$$

**Figure 12:**
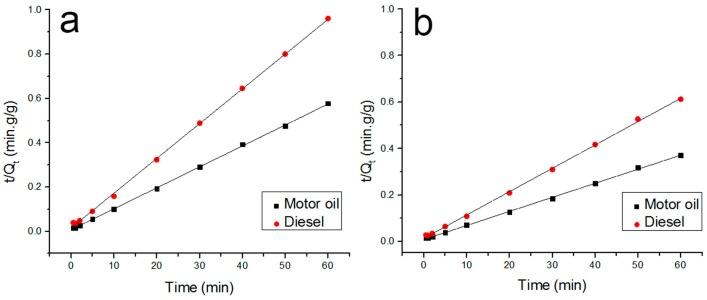
Linear fitting curve of the second-order kinetic law of the Lagergren model ((**a**) raw PLA fiber; (**b**) (8% H203)/PLA fiber).

The saturated sorption capacity *Q*_e_ and the sorption rate constant *K*_2_ can be calculated based on fitting Equation (6). The sorption time *T* for equilibrium can be calculated also as the time for the sorption capacity to reach 95% of *Q*_e_. The linear fitting curves of second-order kinetic law of Lagergren model are shown in Figure 12. All the parameters of oil sorption kinetics are shown in Table 4. The determination coefficients *R*_2_^2^ are well above 0.99, and there is a great agreement between experimental and theoretical oil sorption values, verifying that the second-order kinetic law of the Lagergren model can perfectly describe the sorption process of the prepared fibers for both oils.

#### 3.5.2. Oil Sorption Thermodynamics

Based on the theory of thermodynamics [30], in an isolated system where energy cannot be generated or consumed, entropy change is the major driving force for the transfer of oil molecules from solution onto solid-liquid interface. The thermodynamic parameters such as the changes in the standard free energy (Δ*G*^0^), enthalpy (Δ*H*^0^), and entropy (Δ*S*^0^) can be calculated by the following expression:

(7) $$\ln K_{d}=\Delta S^{0}/R-\Delta H^{0}/RT$$

where *R* is the ideal gas constant (8.314 J/mol/K), *T* is the absolute solution temperature (*K*), *K*_d_ is the equilibrium constant of sorption, which can be calculated as:

(8) $$K_{d}=C_{s}/C_{m}$$

where *C*_s_ (sorbent) and *C*_m_ (mixture) are the equilibrium concentrations of the oil on sorbent and in the mixture, respectively, which can be determined by a double-beam ultraviolet spectrophotometer (Shimadzu UV-1601, Kyoto, Japan) at a specific wavelength.

Δ*G*^0^ can be obtained by

(9) $$\Delta G^{0}=-RT\ln K_{d}$$

The computed values of Δ*G*^0^, Δ*H*^0^, and Δ*S*^0^ for oil sorption on the melt electrospun fibers are listed in Table 5. The obtained Δ*G*^0^ is negative and its absolute value decreases with increasing temperature, which indicates that the oil sorption process is spontaneous and the degree of spontaneity decreases with increasing temperature. The exothermic process during sorption might be attributed to the adhesion of oil onto the fiber surface. In general, the change in sorption enthalpy for physisorption ranges from 20 to 40 kJ/mol, while the chemisorption is in the range of 80 to 400 kJ/mol. The sorption cannot be categorized as single physisorption or chemisorption as the obtained Δ*H*^0^ ranges from −46.3 to 59.2 kJ/mol. Therefore, the oil sorption is mainly physisorption supplemented by a certain degree of chemisorption. The negative Δ*S*^0^ indicates that there is an affinity between the oil and the melt electrospun fibers and the randomness of the oil molecules is deceased at the solid-solution interface.

## 4. Conclusions

Biodegradable PLA fibers have been prepared by melt electrospinning which has potential for mass production. The addition of HBPs to the linear PLA polymer melts successfully improved its fluidity, thus making the reduction fiber diameter possible. The addition of 8% HBPs results in fibers as thin as 896 nm. The feasibility of melt electrospun PLA fiber and PLA/HBP composite fiber employed in oil-water separation was also investigated. It was found that the water contact angle of the prepared fiber and the sorption of oil onto the fibrous mat increases with a decreasing fiber diameter. The highest capacities of oil sorption are 159, 118, and 96 g/g for the motor oil, crude oil, and diesel in a pure oil system, respectively. The reusability and recoverability test demonstrates a drop of about 33%–43% of the original oil sorption capacity and a margin of 98% recovered oil after six cycles of reuse for the electrospun fibers. The obtained data reveals that the oil sorption kinetics agree very well with the pseudo-second-order kinetic model and the sorption process is spontaneous and exothermic. In conclusion, large-scale fabrication of ultrafine fibrous sorbent with a high oil sorption capacity, superhydrophobicity, excellent reusability, and environmental friendliness by melt differential electrospinning technique provides a new approach for the protection of aqueous ecosystems.

## Figures and Tables

**Table 1:** Properties of experimental additive HBPs.

Additive type	Hydroxy group number	Hydroxy value (mg·KOH/g)	Molecular weight distribution from GPC	Experimental molecular weight
H201	6	370	1.15	920
H202	12	260	1.09	2500
H203	24	240	1.18	5500

**Table 2:** Properties of tested oil.

Oil type	Density (g/cm^3^)	Viscosity (mPa·s)	Manufacturer
Motor oil	0.883	209.36	China National Petroleum Corporation (Beijing, China)
Crude oil	0.862	131.41	Petrochina Daqing Oilfield (Daqing, China)
Diesel	0.801	9.54	China National Petroleum Corporation

**Table 3:** Parameters of oil sorption kinetics of first-order kinetic law of the Lagergren model.

Fiber type	Oil type	Fitting equation	Experimental sorption capacity (g/g)	Theoretical sorption capacity (g/g)	Sorption rate constant *K*_1_ (g/(g·min))	Correlation coefficient *R*_1_^2^
Raw PLA fiber	Motor oil	$\ln(Q_{e}-Q_{t})=3.3011-0.0500t$	105.0	27.1	0.0500	0.6859
	Diesel	$\ln(Q_{e}-Q_{t})=2.7496-0.0405t$	61.5	15.6	0.0405	0.4204
(8% H203)/PLA fiber	Motor oil	$\ln(Q_{e}-Q_{t})=3.9928-0.0568t$	159.0	54.2	0.0568	0.7148
	Diesel	$\ln(Q_{e}-Q_{t})=3.4517-0.0512t$	95.5	31.6	0.0512	0.6630

**Table 4:** Parameters of oil sorption kinetics of the second-order kinetic law of the Lagergren model.

Fiber sample	Oil type	Fitting equation	Experimental sorption capacity (g/g)	Theoretical sorption capacity (g/g)	Sorption Rate constant *K*_2_ (g/(g·min))	Correlation coefficient *R*_2_^2^
Raw PLA fiber	Motor oil	$\frac{t}{Q_{t}}=0.00945t+0.00756$	105.0	105.8	0.0118	0.9997
	Diesel	$\frac{t}{Q_{t}}=0.01569t+0.01582$	61.5	63.7	0.0156	0.9994
(8% H203)/PLA fiber	Motor oil	$\frac{t}{Q_{t}}=0.00605t+0.00799$	159.0	165.2	0.0046	0.9994
	Diesel	$\frac{t}{Q_{t}}=0.01005t+0.01308$	95.5	99.5	0.0077	0.9992

**Table 5:** Thermodynamic parameters for sorption test of raw PLA fiber and (8% H203)/PLA fiber.

Fiber sample	Oil type	Temperature *T* (K)	Oil sorption capacity *Q* (g/g)	Standard free energy Δ*G*^0^ (kJ/mol)	Enthalpy Δ*H*^0^ (kJ/mol)	Entropy Δ*S*^0^ (kJ/mol/K)
Raw PLA fiber	Motor oil diesel	293	114	−17.5	−58.7	−0.141
		303	105	−16.0		
		313	101	−14.6		
		293	70	−12.8	−46.3	−0.114
		303	62	−11.8		
		313	59	−10.6		
8% H203/PLA fiber	Motor oil diesel	293	167	−20.3	−59.2	−0.133
		303	159	−18.9		
		313	155	−17.6		
		293	104	−14.8	−47.1	−0.110
		303	96	−13.6		
		313	94	−12.9		
